# “Blood is thicker than water” - experiences and perspectives of family caregivers of people living with severe mental illness at Holy Water traditional healing sites, Addis Ababa, Ethiopia

**DOI:** 10.3389/fpsyt.2024.1495058

**Published:** 2025-02-21

**Authors:** Yonas Baheretibeb, Dawit Wondimagegn, Lisa Andermann, Samuel Law

**Affiliations:** ^1^ Department of Psychiatry, School of Medicine, Addis Ababa University, Addis Ababa, Ethiopia; ^2^ Department of Psychiatry, Faculty of Medicine, University of Toronto, Toronto, ON, Canada

**Keywords:** Ethiopia, family caregivers, traditional medicine practitioner, Holy Water, intersectoral collaboration, mental illness, persons with psychiatric disorders

## Abstract

**Background:**

Family caregivers of people with severe mental illness (SMI) are the backbone of the mental health care system in resource-limited family centered cultural setting like Ethiopia. This exploratory qualitative study examines the experiences and perspectives of family caregivers at two Ethiopian Holy Water treatment sites for people with SMI in Addis Ababa, where a collaborative project exists between traditional healers and biomedical practitioners.

**Methods:**

Eleven family caregivers at two Holy Water treatment sites in Addis Ababa, Ethiopia were interviewed in 2021, using a semi-structured interview guide. The transcribed material was analyzed using qualitative thematic analysis. Inclusion criteria for study participants were over 18 years of age, capacity to give informed consent, and has a family member with mental illness for whom the family caregiver has sought help at the Holy Water treatment sites and at the collaborative Clinic.

**Results:**

Content analysis found seven notable themes: 1. Strong sense of obligation and responsibility and ongoing provision of care; 2. Caregiving puts a serious strain on caregivers’ lives and established family roles; 3. Chronicity and persistence of illness take toll on family caregivers and networks of support; 4. Family caregivers appreciate the supportive religious setting and attendants at Holy Water treatment community; 5. Family caregivers develop a community of mutual support for each other; 6. Severe shortage and poor access to formal biomedical services and appreciation of the collaborative Clinic; 7. Burden, exhaustion, and loss of hope regarding the future.

**Conclusion:**

The study shows that families in Ethiopia face a protracted and heavy caregiver burden in their caretaking duties, often in isolation, with a severe lack of formal biological treatment and psychosocial support. Informal assistance and mutual support form part of the culturally shaped support networks, but there are on-going challenges. Innovative programs with collaborative approach show some promise. More development of community mental health services and support are urgently needed.

## 1 Introduction

People with severe mental illness (SMI) suffer significant emotional, cognitive, and physical functioning impairments that often leave them unable to take care of themselves (1, 2). They often need day-to-day support, and this duty and responsibility falls mostly on their close family members (3). Globally, family caregivers are typically the backbone of a community care system, taking on the primary responsibilities in caring for those with SMI, along with whatever the community and mental health system may provide (4–6). This is particularly true in low and middle income countries where community resources are limited and treatment gaps – referring to the disparity between those who need mental health care and those who actually receive it from formal or biomedical based services - are large (7–9).

It is estimated that one in four households in the world have a member who has mental or behavioral issues that requires some form of care by families (10–12). As family caregivers struggle to balance caregiving and living their own lives, their physical and emotional health are often jeopardized. Family caregivers’ burden in terms of chronic stress, stigma, guilt, helplessness, psychosocial and vocational loss, physical strain, relational pressure, and financial cost are well known global issues (13–16). Many caregivers may also develop their own mental health problems, commonly in the form of depression, and/or anxiety (16–18). Family caregiver burden is a multi-dimensional concept but captured vividly by an eminent psychiatrist who did much caregiving to his wife who suffered from Alzheimer’s disease: “Caregiving is not easy. It consumes time, energy, and financial resources. It sucks out strength and determination…. It can amplify anguish and desperation. It can divide the self. It can bring out family conflicts. It can separate out those who care from those who can’t or won’t handle it. It is very difficult.” (19).

Due to inadequate resources and mental health infrastructure, family caregiver burden is considered even more entrenched and a silent problem in the developing world setting (20). Furthermore, recent changes in family structures and socio-economic upheavals in many parts of the developing world have posed additional threats to this system of informal care (21). The interactive and vicious cycle of how severe mental illness impacts on quality of life of the patient, and in turn that of the caregiver, and in turn the family as a whole is tremendous (22).

In Ethiopia, a developing country in East Africa of more than 126.5 million (23), the mental health care infrastructure is among the lowest in the world, according to the World Health Organization’s Mental Health Atlas (24). Research shows, for example, for people with schizophrenia, the treatment gap for those who could have benefitted from biomedical treatment but never received any was 90% (25); for those who did eventually receive psychiatric care, the average “delay” in help seeking was around 38 weeks (26). However, is place of formal care, the most common form of “alternative” treatment for those with SMI and other mental illnesses is through religious healers who use Holy Water. Holy Water is believed to embody the spirit of Christ that can exorcise evil spirits whose possession on the person is thought to cause the illnesses (26, 27). Holy Water treatment is administered by Orthodox Christian priests, and attended by people of that faith (43.5% of the population), but also by others of different faith and the general population at large due to its popularity and a culturally shared illness explanatory model (28–30). Moreover, studies have shown that the majority of Ethiopians prefer traditional and alternative healing to biomedical services for mental disorders - more than 90% of first encounters in mental health help seeking is with traditional practitioners such as Holy Water healers (31–33). In addition, they also prefer informal forms of help, such as family and friends, over those of the biomedical health centers for their major illnesses (34, 35).

The reasons for the overwhelming preference for traditional healers in Ethiopia is complex, and it is likely related to - like many other developing country settings - a lack of availability and infrastructure of biomedical treatment, plus historical, cultural, economic, and illness stigma reasons (30, 36–39). Recognizing the prevalence and power of traditional healing, one of the efforts to scale up mental health services is to promote collaboration between that and biomedicine (40–42), with some models of success in Nigeria (43), Kenya (44–46), and South Africa (47), for examples.

In Ethiopia, we have recently reported a successful collaborative clinic between biomedical psychiatry and two traditional Holy Water religious healing sites in Addis Ababa (48). In this innovative collaborative model, we held leadership meetings, a series of consultation workshops, mutual visits to Holy Water healing sites and psychiatric hospitals, and created a Clinic close to the two Holy Water treatment communities, among others. Through this collaborative process, we had the opportunity to learn the experiences and perspectives of family caregivers who play a vital and indispensable role in the overall caring of those with SMI.

Quantitative research on family caregiver burden in the care of people with SMI in Ethiopia has shown a considerably high level of burden, particularly related to personal emotional toll, lack of stability, and financial costs, among others (49–51). Few qualitative studies are available to understand the in-depth, lived experiences of families who care for family members with SMI, with particular attention to their on-going experiences, narratives, values, expectations, and coping mechanisms. Furthermore, there is no study conducted in low-and-middle-income-country settings that explored caregivers’ experiences at the intersection of working with both traditional healers and biomedical sciences. The purpose of this study was to evaluate such experiences of family caregivers of patients presenting at a collaborative site where Ethiopian Orthodox Holy Water Healers and psychiatric clinicians collaborate.

## 2 Methods

### 2.1 Study sesign

This qualitative study was informed by constructivist grounded theory to explore this little known area to generate understanding rooted in data, and assumes a relativist approach, acknowledging multiple perspectives and realities of both the study participants and the researchers who are asking the questions (52). The methodology facilitates the in-depth, nuanced, contextual and sensitive exploration of unique cultural and social determinants that may influence perception, attitude, and behaviour of the participants (53). Based on this research framework, a literature review, and local expertise in working with the study population, the research team developed a semi-structured interview that aimed to explore these qualitative aspects on their role as caregivers for their family members with mental illness. The semi-structured interview questions were broadly informed by cultural psychiatry regarding explanatory models (54), mental health literacy literature (e.g. 55), and family care-taker burden (e.g. 56). The interview also paid attention to “sensitizing” concepts related to the participants’ care-taking experiences (57). Thematic analysis was performed on the data collected from the interviews (58).

### 2.2 Study sites, recruitment and sampling

The study sites were two Holy Water treatment communities located close to two Ethiopian Orthodox churches– St. Michael’s and St. Mary’s - in the Entoto suburban area of Addis Ababa. The treatment communities together were comprised of approximately 1000 individuals, including patients, their attendants, family members and the priests and their assistants. The patients reside in simple dwellings near the two churches, supported by their attendants and their family members. Both churches have a natural supply of spring water that has been considered Holy through church ordination. At designated treatment facilities, Orthodox priests use Holy Waters to perform various forms of spiritual cleansing and exorcism, and religiously based counseling. These services are free. Families may choose to donate to the church. The collaborative Clinic between Holy Water treatment sites and biomedical practitioners was created in 2012, and located close to both the Holy Water communities; referrals to the Clinic were made by the priests, attendants and family members (48).

Family caregiver participants were recruited at the collaborative Clinic. Inclusion criteria for study participants were over 18 years of age, capacity to give informed consent, and has a family member with mental illness for whom the family caregiver has sought help at the Holy Water treatment sites and at the collaborative Clinic. Family members who accompanied the patients seeking care were approached for recruitment. Participants were reassured that participation or not in the research did not affect the treatment of the patients. A purposive sampling approach was used in selecting family caregiver participants to maximize an informative and representative sample (59, 60).

### 2.3 Data collection

The semi-structured interview guide included suggested opening remarks and questions, with a range of sensitizing concepts reminded and embedded in the questions that also promoted logical and ease of flow and possibilities to pursue any new and unexpected content. The following is a list of the questions that explored the topics related to the current study (all questions have prompts to explore details when appropriate): *What barriers do you encounter when seeking mental health services?* (Assessed access to mental health services, and subtopics of reasons to come to Holy Water treatment, and Clinic, advantages and disadvantages of either, availability and quality of mental health facilities, experiences and challenges faced during psychiatric hospitals and Holy Water treatment, financial constraints and transportation issues, etc.). *How do you perceive your role in providing care at Holy Water sites?* (Assessed role of family caregivers, and subtopics of daily responsibilities and emotional toll, spiritual and cultural beliefs influencing care, transition or roles as caregiver, sense of responsibilities, how daily life and well-being were affected in terms of relationships/work/school/finance, etc.). *In what ways has caregiving at Holy water altered family roles and relationships?*(assessed impact of caregiving on family dynamics, and subtopics of changes in family roles and responsibilities, impact on relationships with other family members, emotional and psychological effects on caregivers, and reflect on how caregivers felt in their roles). *How has the caregiving experience affected your social connections?* (Assessed changes in social support networks, and subtopics of support from friends and community, changes in social activities and community engagement, emotional and psychological consequences of caretaking). *What suggestions, hope, and concerns do you have about the future?* (assessed future worries and related issues, and subtopics of concerns about the well-being of their loved ones, personal health and ageing, alternative care options and support systems, current coping The interviews encouraged and facilitated rich descriptions of individual experiences and pursued sensitized and related areas of inquiry based on the main research question (61).

A total of eleven (11) participants were recruited and participated in the study. The study interviews were conducted in January-June 2021. The interview guide was translated into *Amharic*, the most common local language. The interviews were coordinated and conducted in *Amharic* by a research assistant (RA) who has a sociology background, local language proficiency, experience in qualitative research, and had previously worked at a Holy Water site. Prior to conducting the interviews, the Principal Investigator (PI) trained the RA on the main objectives of the study and the semi-structured interview methodology. The information was gathered anonymously and without personal identifying information to optimize candidness. The interviews began with open-ended questions to foster rapport building and reflection. The interviews were all single session, lasting about 30-50 minutes, held at a private location convenient to the participant. Interviews were tape-recorded and supplemented with field notes. After each interview, the first author (YB) reviewed the case and field notes and discussed initial impressions with the RA and resolved questions and made minor amendments to the interview guide as the study progressed (53).

### 2.4 Data analysis

Interviews were transcribed from *Amharic* into English by the same RA who conducted the interviews. The RA checked for the accuracy of the transcripts and ensured preservation of important local language concepts and nuances. When in question, the RA consulted the PI and the research team for input. Overall, there were no significant interview, data gathering and transcription issues. A selection of the English translations was compared against the original *Amharic* transcripts by the PI (the first author, a psychiatrist with local language proficiency and broad clinical and mental health system and research experiences). The research data was then analyzed using a thematic analysis approach (58). Some basic “sensitizing” concepts -such as family responsibility, financial strain, relationship changes - were used to inform initial thematic coding, while broader and new content and concepts were actively captured. The RA and the first author independently coded and made memos and generated categories and themes on seven of the eleven English transcripts. The coding and memos from both were reviewed and compared and then consolidated to enhance the completeness and consistency of the analyses. Any significant difference was discussed and resolved by reaching a consensus. All differences were minor and resolved. Notes on the discussions were also taken to inform the ongoing coding process. As analysis proceeded, earlier transcripts were re-read and re-coded as necessary to allow the re-capturing of newly emerging themes and concepts. Frequent and salient codes were identified, and thematically linked codes were clustered to generate distinct and informative thematic categories. The remaining four English transcripts were then coded by the first author using the final, agreed-upon coding framework generated from the previous seven transcripts. Data saturation was determined by the RA and PI when no more new codes or categories emerged – this occurred after about seven of the eleven full interviews, ensuring the fullness of data (53). Quotations were selected to represent themes in the data.

### 2.5 Ethical approval

For each interview, the RA explained the purpose of the study, answered any question, explained the consent process, and formally gained permission to proceed. The study followed the institutional ethical guidelines involving human subjects and obtained full written consents for interviews and recordings. The study received approval from the Ethics Board of the Addis Ababa School of Medicine in September, 2020.

## 3 Results

### 3.1 Socio-demographic characteristics of the participants/caregivers

Of the eleven participants, nine were male, two were female; most (*n* =9, 81.8%) were married, two were single. The ages of the participants ranged between 18 and 59. The majority of the participants were employed, and Orthodox Christian. Of the participants’ eleven family member with mental illness, ten were taking both biomedical and religious treatment, one was taking only Holy Water treatment (but the family member caregiver visited the Clinic for help). (Please see **Table 1** for demographic details).

**Table 1:** Family caregiver participant social-demographic details.

	Participants/Family Caregivers (n)
Marital Status	
Single	2
Married	9
Gender	
Male	9
Female	2
Age group	
18-29	4
20-39	5
40-59	2
Employment status	
Employed	7
Unemployed	4
Religion	
Orthodox -Christian	8
Protestant-Christian	2
Muslim	1
Level of education	
No formal education	–
Primary education	2
Secondary education	9
Post-secondary education	–
Duration of family member with mental illness (years)	
0–10	9
11–20	2
21–30	–
Relationship of the participant to the patient	
Parents	4
Siblings	5
Spouse	1
Relative	1
Period of Holy water community stay (months)	
0-10	2
11-20	7
21-30	2

Thematic analysis of the participant’s caretaking experiences produced seven major themes, as follows:

#### 3.1.1 Strong sense of obligation and responsibility and ongoing provision of care.

In Holy Water communities, family members were still the main caregivers for those with SMI, while some patients were also helped by hired attendants. The family caregivers took care of the mentally ill relative’s daily needs, including making meals, taking them to the priests for treatment and spiritual support, monitoring their care, security, physical safety, and signs of violence. The work was intensive, and their commitment serious.

One participant (F.2 male, age 39) stated:

*“I’ve been tirelessly taking my sister to countless holy water sites ever since her illness began. It breaks my heart that I’ve had to spend every penny I saved for my retirement on their care. I’ve even resorted to selling my belongings just to keep up with the expenses.*”

Another participant (F.8, female, age 31) reported:

*“I’m doing everything I can to keep my father safe and help him find peace. It’s been incredibly difficult, but I’ve had to resort to restraining him and bringing him to Holy Water treatments for his well-being. I’ve been by his side every step of the way, cooking for him, bathing him, and doing everything I can to make sure he gets the care he needs. It’s a heavy burden, but I’ll do whatever it takes to help him through this.”*

One other participant (F.5, male, age 39) stated that:

*“I have been with him every step of the way, at home and in the hospital, and I will continue to stand by his side in the future. As we go through the treatments at Holy Water, I will pour my heart and soul into providing him with all the support and care he needs. He is my everything, my top priority. I truly believe that God has entrusted me with the care of this precious, mentally ill child for a reason. I will do whatever it takes to help him find peace and healing.”*

#### 3.1.2 Caregiving puts a serious strain on caregivers’ lives and established family roles

In providing a wide range of care and encountering many situations and challenges, family caregivers reported strain and interferences with their capacity to perform other everyday duties, including looking after oneself, and taking care of other family members.

One participant (F.2, male, age 39) stated:

“I used to be the major provider for the family, but recently I’ve been living with my mentally unstable sister and traveling to holy sites while leaving my wife, three children, and job behind. Only God is aware of how they survive.”

One family participant (F.10, male, age 23) said:

*“At home, my mentally ill son used to assist me in cooking and dish washing but now I have to cook and transport food for him almost every day to these Holy water sites, it is not an easy task.”*

Another Participant (F.1, male, age 45) described a role shift:

*“I was the family’s primary provider at the start of the illness, and I was employed full-time. I eventually had to miss work, forced to take my brother to the hospital frequently and start working part-time. However, I have since completely given up working, moved in with him to Holy Water, and am now financially dependent on others.”*

The family caregivers’ lifestyle or role often had to change to accommodate the caregiving role. One participant (F.5 male, age 39) stated:

*“I feel like I need to make a drastic shift in my life since my kid is dealing with a mental condition. It was hard for me to give up the time I used to spend alone; I had to bid farewell to close friends, let go of a loving partner’s devotion, give up the coziness of reading literature I liked, and forfeit the thrill of watching exciting sports. Even though it left me feeling empty and alone, I knew in my heart that raising my son was what mattered most.”*

Some participants reported that their relationships with their other immediate family members deteriorated over time because of the stress and difficulties of caring for mentally ill relatives in Holy Water sites.

One participant (F.7, male, age 28) reported:

*“I spend all my time taking care of my cousin at the Holy Water, which leaves me unable to go to funerals for my close relatives or comfort sick and imprisoned family members. It hurts a lot that my own family doesn’t understand how hard it is for me and doesn’t realize the sacrifices I make. I feel very alone and wish they could understand and support me.”*

Due to the strain these relationships placed on them, some of the participants’ long-term marriages ended and families disintegrated. As one participant (F.11, male, age 59) reported:

*“I spend all my time taking care of my first wife’s child, who has intellectual disabilities, at Holy Water. Unfortunately, my second wife doesn’t visit me often because she doesn’t understand the situation. I was completely shocked and couldn’t believe it when I heard the sudden news that she wanted a divorce. It has made my second marriage uncertain and difficult, and it has deeply affected me emotionally. I feel very sad as I try to accept that the strong connection we once had is now in serious danger.”*

One participant (F.10, male, age 23) also reported:

*“My life has been utterly flipped upside down by caring for my mentally ill son. Among the difficult choices I had to make were selling my cattle, renting out my farm, and sending my kids and my wife to live with their grandparents. My own house seems empty and lonely as a result. My wife’s own illness hasn’t improved as much as I had anticipated, despite my sincere attempts to find comfort by traveling to several Holy Water sites.”*

Another participant (F.7, male, age 28) said:

*“I have been looking after my cousin for the past eleven months, so I was shocked when my wife unexpectedly left and traveled to one of the Arab nations without notifying me or anybody else. Our four children were left behind in an empty house by her. I’m concerned that my kids could become homeless. It fills me with despair.”*

#### 3.1.3 Chronicity and persistence of illness take a toll on family caregivers and networks of support

Treating mental illness at Holy Water sites often takes a long time, with no end date in view. Many family caregivers spoke of the gradual disintegration of their social support networks which were made up of nuclear and extended families, friends, and community members. The support was more available at the beginning of the treatment, functioning as a dependable source of moral and emotional support, and providing patients and families with tangible necessities such as food, clothing, and money. However, with time, the support tended to decline.

One of the participants stated (F.1, male, age 45):

*“Friends, neighbors, and members of the community came to see us when my brother was ill and when we initially moved to Holy Water. They supported us emotionally in addition to bringing us food and cash. The folks in the community helped us out by cooking and providing meals even after we relocated to Holy Water and out of the city. They were of great assistance, and I appreciated their aid. But as time passed, I stopped getting such assistance.”*

The persistent nature of the illness and the associated stressors also result in family caregivers gradually becoming more alone in their caregiving and feel stranded with needs. One participant described (F.4, female, age 37):

*“As soon as I brought my brother to the Holy Water sites for treatment, people from the village and my close family started coming to see me frequently, but they gradually stopped when they saw the ailment was permanent. I don’t blame them for being tired out; they must have been. They might be dealing with issues of their own. I appreciate all of their outstanding assistance.”*

Another participant (F.11, male, age 59) reported about families sometimes are forced to give up:

*“With a heavy heart, I stayed at this holy water place for nearly two long years. Families, filled with hope, would bring their young children here seeking treatment. However, as the months passed, these families would be drained both financially and emotionally, and their strength would wane. It was a heartbreaking sight to witness, as they ultimately made the heart-wrenching decision to abandon their loved ones and depart from this place. I cannot help but feel a deep sorrow as I believe this Holy Water place becomes a tragic source for mentally ill individuals who are left to wander the streets, lost and forgotten.”*

Elderly members of the family are key to the support network – their support carries respect, credibility, cultural approval, sets an example for other members of the community, and helps to organize resources, such as food and money. Elders were typically involved at the beginning of the illness and treatment when they are informed of the patient’s diagnosis and situation. They were also consulted on what treatment to use and which Holy Water places to visit. However, this important resource is also tested by the chronicity of illness. One participant described (F.8, female, age 31):

*“When the illness first started, I told the family elder when my father started destroying household items. He called a family meeting to discuss what to do; he contacted Holy Water priests to help me at the Holy Water sites; and he also asked the women in the village to help contribute dry food to help us survive during our stay at Holy Water. However, the longer we stay, the less I get this support”.*

Another Participant (F.6, female, age 34) reported:

*“I feel a deep sadness as I recall the story of a family member from a rural area who is taking care of his father. We live in the same house, and one day he asked me to watch over his elderly father, who is mentally ill, for just two weeks. He needed to travel back to his home village to bring money and dry food. However, it has been six months now, and I haven’t heard anything from him. I don’t even know his address. As a result, I find myself shouldering the responsibility of caring for both his father and my sister. The weight of this situation fills my heart with profound sorrow”.*

#### 3.1.4 Family caregivers appreciate the supportive religious setting and attendants at the Holy Water treatment community

Some participants reported how they appreciate the spirituality in the care at Holy Water sites, and their experience positively impacted their perspective on their religion. One of the participants (F.9, male, age 18) described:

*“I used to believe in liberal Christianity, but something significant happened that changed my perspective. When I saw the unwavering spiritual support and limitless love given to my father, who suffers from severe mental illness, at the Holy Water site, it shattered my previous beliefs. In that moment, my faith underwent a profound transformation, and I embraced a more conservative outlook. Now, I have a strong sense of certainty and unwavering dedication to my faith.”*

Another Participant (F.2, male, age 39) reported:

*“The spiritual service I am getting from religious healers in the church has helped me develop resilience in the face of stress to care for my sister. I have a sense of perspective that enables me to navigate stress with resilience, hope, and a belief in my ability to overcome challenges. I am transformed…”*

Another participant (F.10, male, age 23) reported:

*“During my stay at the Holy Water location, I had a genuinely remarkable event. A woman lost her belongings, which caused her to become extremely anxious and even attempt self-harm. Nonetheless, she fully recovered under the frequent supervision and counsel of the Holy Water priest. She now pays us sporadic visits, indicating that her condition has really improved.”*

Several participants highlighted the assistance they received from Holy Water attendants. Having help enabled family caregivers to have time so they could continue to work and support other family members. One Participant (F.4, female, age 37) stated:

*“Holy Water attendants help me take care of my brother while I work five days a week for a minimal charge, which is a huge assistance because I have five children at home and it is hard for me to not work to feed them.”*

Participant (F.4, female, age 37) also reported:

*“Our days at Holy Water begin early in the morning as I take my brother to Holy Water treatment near the hill. After having breakfast, the Holy Water attendants arrange various activities for us, like playing soccer or card games. They make sure our day is enjoyable and not boring. We are truly grateful to them for their efforts.”*

Some participants appreciated the skill of Holy water attendants in handling agitated patients. One participant (F.7, male, age 28) described:

*“The only people who could restrain my cousin’s physical aggression when he was incredibly upset and aggressive towards everyone else were the Holy Water attendants. Without Holy Water attendants, I couldn’t see myself taking care of my baby. They vocally and dramatically soothe him down without ever touching him.”*

#### 3.1.5 Family caregivers develop a community of mutual support for each other

Sharing the same problem or experiencing stress together can create a strong sense of connection and foster a supportive environment, similar to a family. One participant (F.3, male, age 29) reported:

*“In this place, we are facing similar challenges and stressors; as a result, we empathize with and understand each other’s experiences on a deeper level. This shared understanding can create a sense of camaraderie and a safe space for us to express our emotions and concerns without fear of judgment.”*

Another participant (F.4, female, age 37) reported:

*“While I’m gone at the hospital, other family members staying at Holy Water are taking care of my mentally ill brother. This shared experience of having a loved one with mental health challenges can foster a strong sense of camaraderie among family members. It creates an environment where they may support and understand one another, much as in a close-knit family.”*

Another participant (F.7, male, age 28) reported:

*“The Holy Water community faces the same stressors; usually, we provide support to one another. This support comes in various forms, such as offering advice, sharing coping strategies, sharing food, or simply lending a listening ear. For me, just knowing that others at Holy Water are going through the same difficulties provides me with a sense of validation and comfort.”*

One participant also (F.9, male, age 18) stated:

*“It gives me a feeling of identification and belonging to be a part of a group that is dealing with the same challenges. I feel a connection to other caregivers who have similar experiences and strive to overcome hardships. I received emotional support from this sense of community, which increased my resiliency and encouraged me to fight and not give up.”*

#### 3.1.6 Severe shortage and poor access to formal biomedical services and appreciation of the collaborative Clinic

One of the reasons family caregivers use the Holy water sites is their ease of access. Several family caregivers had struggled to access formal or biomedical mental health care due to a lack of such services, and a lack of social support in general. Due to the unpredictable nature of the illness, it was often difficult and sometimes impossible to use formal biomedical care. When available, some could not afford the costs and travel the distance associated with seeking care, as explained by one participant (F.1, male, age 45):

*“If your family has enough money, they will take you to Amanuel Psychiatric Hospital so that you can receive the care and support you need. However, if your family doesn’t have enough money, you might be left alone on the streets, or they might try traditional methods like using Holy Water to help you. This shows that some people have better access to mental health care than others, and it’s important that everyone receives the support they need, regardless of their background or circumstances.”*

One participant (F.7, male, age 28) echoed:

*“The journey to the hospital from my village feels like an endless trek, taking around eight grueling hours on foot. The thought of hiring a taxi is daunting as it is exorbitantly expensive, and to make matters worse, some heartless drivers may even refuse to transport severely mentally ill individuals. It’s a painful reality that adds another layer of hardship to an already difficult situation.”*

Another participant (F.3, male, age 29) stated:

*“The hospital and the police don’t have an ambulance service to come and pick up my brother when he’s going through a severe mental illness. It’s tough for us every time we need to move him during these times. I have to spend twice as much money on his transportation to Holy Water, which makes things even harder. I’m not sure who is supposed to help us in these desperate situations. It feels like I’m the only one going through this extremely difficult and terrible struggle.”*

Overcoming these challenges and making their way to a psychiatric facility, participants often found that the closest institution is ill-equipped, with poor infrastructure and psychosocial support to offer the patients the treatments they required. Sometimes the hospitals or medical facilities did not provide medicine for patients, or they didn’t have beds to manage the crisis, etc. As stated by one participant (F.8, female, age 31):

*“I was terrified when I had to take my father to the psychiatric hospital. I begged the medical staff to admit him and give him the help he desperately needed, but they said there were no beds. After just one injection, they sent him back home with me. It felt like we were abandoned in our time of need.”*

One participant (F.2, male, age 39) also reported:

*“I gave everything to help my sister through mental health relapse, pouring my heart, soul, and money into it. It was a draining journey, but I did it out of love and desperation to see her get the help she needed. After all that, the healthcare professionals prescribed a medication that wasn’t available anywhere. It felt like a gut-wrenching blow, a cruel twist of fate after everything we had been through.”*

Another participant (F.5, male, age 39) stated:

*“Taking my son to the hospital for his mental health and substance abuse issues was a difficult decision, but I believed it was the best option for him. However, after he was admitted, I was surprised to discover that the hospital lacked a rehab program or engaging activities for the patients. Instead, it appeared that they primarily relied on medication to sedate them. Witnessing my son in this condition without any explanation was disheartening, especially after everything we had endured.”*

Participants also spoke of how receiving free biomedical care at the collaborative Clinic on Holy Water site reduced their burdens; as one participant (F.1, male, age 45) reported:

*“I used to spend quite a bit of money on travel and medications for my brother when we made our monthly journeys to the hospital. I feel much better now that I am getting the medication and therapy for free without having to go to the hospital this motivated me to do more.”*

The same participant (F.1, male, age 45) also reported:

*“A religious leader gave me instructions to bring my brother to the outreach Clinic in addition to receiving the Holy Water treatment for a specific reason. Since I trusted their advice, I started to see improvements after his first treatment session. Maybe he’ll recuperate completely and be able to go back home.”*

Another participant (F.8, female, age 31) said:

*“Even though I am completely drained, both physically and emotionally, from tending to my father day and night, I will push through with unwavering determination. I refuse to give up because I cling to the hope that the Holy Water and medication might just be the answer we’ve been desperately seeking. I will keep going, no matter what, for the chance of bringing about a positive change in my father’s condition.”*

Another participant (F.6, male, age 36) stated:

*“A Holy Water priest gave me advice to obtain my sister a tablet from the outreach Clinic and give it to her with Holy Water. She surprised me by showing some progress after doing it. I will continue to do it.”*

Another participant (F.11, male, age 59) reported:

*“Having the service available at the Holy Water location is a wise proposal. Nevertheless, the outreach Clinic only comes to us once every two weeks and is not open every day. We have to wait for them to arrive, which makes situations tough for us.”*

#### 3.1.7 Burden, exhaustion, and loss of hope regarding the future

Family caregivers became isolated and lacked moral, emotional, financial and physical support as social support networks gradually vanished. They realized that they were more alone over time, facing no end to a chronic illness. As one participant (F.10, male, age 23) observed:

*“During my time at various Holy Water centers, I saw many rural family caregivers who were unable to bear the financial strain and left their relative patients behind, vanishing in the middle of the night, leaving the patient homeless. I have no choice but to embrace him since he is the one person that God has given me to care.”*

In the context of a pervasive lack of help, the caregivers were additionally concerned about who would take care of their loved ones when they were no longer able to do so. Elderly caregivers were especially impacted by this problem since their worries for their children’s futures after their own deaths gave them a great deal of stress. One participant (F.10, male, age 23) reported:

*“I go to church frequently and spend a lot of time praying, reflecting, and sobbing. What will happen to my mentally ill son in the future? If this Holy Water location is unable to heal him, who will transport him to another one? Will he improve or deteriorate? What if I pass away right now? Who will look after him? Or will he become a vagrant like others?”*

Another participant (F.5, male, age 39) described:

*“I am aware that blood is thicker than water. Yes! I am his only caregiver. Who else could? I have been responsible for taking care of him since I brought him into this world. My biggest concern right now is becoming old and becoming feeble and forgetful since I don’t know what will happen in the future.”*

## 4 Discussion

The current qualitative study shows that families of people with SMI play vital roles in caretaking. Driven by a strong sense of responsibility and obligation, their presence in traditional Holy Water healing sites reflects socio-culturally ingrained practice, and a severe shortage and poor access and quality of biomedical alternatives. The family caregivers face detrimental changes in their personal and family lives, dwindling support over time, and caregiver burden with increasing helplessness and hopelessness. Some systems of support, including paid care and mutual aid, are available, but far from adequate. More community mental health services and support are urgently needed. An innovative approach in collaboration between available modalities of care in such a resource-limited setting holds some promise.

Our findings highlight the sociocultural reality in many low and middle income country settings such as Ethiopia that most people seek health care, especially mental health care, in traditional healing settings as their first choice (36, 62). Research has shown that these help-seeking patterns are associated with high socio-cultural acceptability and compatibility with prevalent illness explanatory models, easy availability, relative affordability, lower levels of stigma related to traditional healing, and often some distrust of the biomedical approach. (46, 63).

What the researched factors do not reflect and often assumed is the availability of family caretaking. This underlying and critical ingredient, as heartily portrayed by the participants, involves the unwavering, dedicated commitment and sense of responsibility families carry for the ill members. Family caregiving of a loved one is universal, while also deeply interwoven with social, economic and cultural conditions (5). Some variability exists, such as a collective, kinship-based social setting may expect and rely more on family caretaking, while more individualist settings are relatively less so (6). Societies with more resources dedicated to community level of care, often in developed counties, may have less sheer reliance on family care provision, with less interruption on the lives of family members (64). In less developed parts of Asia, for example, more than 90% of people with SMI live with families, and in some instances, the family’s caring ability reflects the respectability of the family in the community (65, 66). Similar figure is lower in North America, as cultural and resources factors differ (6, 67, 68). With both a strong collectivist and under-developed setting such as Ethiopia, the extraordinary commitment and self-sacrifice involved in the caretaking as reported by the participants is remarkable, and families are truly the de facto backbone of community mental health care (26).

While the commitment is tremendous, the responsibility and stress on the caregivers can also be extraordinary. The functional, material, financial, and general life burden were very felt in the current study. When families bring their ill members to a Holy Water treatment site, it means that they have encountered a crisis, urgently in need of treatment and help. It also meant that family burden and life impact had started prior to coming to Holy Water sites, and the families had exhausted their capacity to manage at home. Seeking a residential form of care with treatment and assistance being available on site, giving the families some respite is the preferred solution (48). Though not assessed specifically, family caretaking by the participants most likely took on extraordinary toll on their lives, as told by many (49).

Family burden is reflected in many ways; one of the strong themes in this study is a financial one, both in terms of direct cost, hiring help, and lost opportunity of earning. Socio-economic burden reflects historical and current limitations in settings where low or near-non-existing levels of formal, publicly available, community-based support exist for families. The accumulative costs of mental health care in traditional healing sites, as well as clinics and hospitals appear to be prohibitive. All these likely lead to more self-reliance on family caring, creating a self-reinforcing negative cycle of caregiver and their financial burden (22). Overall, caregiver and particularly financial burden is more likely to take root in resource limited settings when compared to settings where formal and community health care services are more available, so caretaking is not solely rested on the shoulders of the family (69).

The participants’ descriptions of having to endure personal and work role changes and when no other options are readily available also reflect the notions of role captivity and role overload (70). These occur when caregivers have to take on all the care duty and feeling trapped within, often having to put their ill family members’ needs above their own (71, 72).

The participants also reported on the inordinate challenges related to the chronicity and enduring need of care by people with SMI. This reality, often not anticipated or met by well-meaning community and extended family, resulted in further social isolation and entrenched burden. It is a challenge even for those who are committed, and it is an under-recognized problem for society at large. Creating support that is sustained over time that matches the nature course and characteristic of SMI is much needed (3, 73).

It is of note that the respondents did not discuss explicitly about stigma of a family member’s mental illness and their impact on them. It is possible that the topic itself was hard to discuss. The participants may have also just assumed that everyone knew it was present. Stigma is prevalent and may be embodied in the participants’ reports on how their progressively limited and waning interactions and engagement they had with the extended family and community (74). Research shows the effects of stigma on mental illness are pernicious and pervasive, associated with families being shunned from social interactions, resulting in social isolation and avoidance of help-seeking (75–77). This could also be further associated with family caregivers’ internalized sense of shame, and self-blame that may contribute to a negative cycle of detachment and sense of loss (71, 78).

In their adversity filled experiences, the participants also reported creative, uplifting aspects in finding solace and appreciation from their religion. Research shows religion and faith-based approach asserts their power of healing through a strong shared cultural understanding of the illness explanatory models, local knowledge of their needs and desires, being less-stigmatized, and having faith in a higher power based on the shared spirituality and or religion (79–81). These capacities are often undervalued and underappreciated in biomedical approach. Recent qualitative studies on religious healing in Ethiopia show that a deep respect for the person is part of the core tenets of the Ethiopian Orthodox Church, potentially translating into an accepting and empathic attitude towards patients and family, and possibly forming the bases of the families’ endorsement of their religion (36).

Family participants also reported creating a more supportive community through mutual aid was helpful, with families helping each other with firsthand experiences in caring for people with SMI. The findings echo those that show mutual aid and informal community networks can be invaluable, especially when formal and established services are lacking (27, 82). Ethiopia has a rich and unique socio-cultural tradition of mutual care – organizations such as *edirs* that provide mutual-help for community members on resource intensive challenges such as funerals, home building, or securing money for emergencies, etc. (83, 84). On Holy Water sites, mutual aid could potentially be very meaningful and burden-reducing, and enabling and strengthening these informal network could be a very promising form of improving mental health care, as some similar organizations in North America have demonstrated (85, 86). However, there remains barriers such as stigma and low acceptance of mental illness for such community mutual support in Ethiopia (82). Using social contact interventions to address stigma and normalize mental illness for more community support are parts of recommendations from the Lancet Commission that focused on tackling mental health stigma and discrimination (87).

Echoing local knowledge and previous research, participants in the current study typically sought out biomedical mental health services after attending traditional healers (63, 82, 88). For those families who would accept a biomedical approach, they lamented remarkably how grossly inadequate, difficult to access, and costly that system is in Ethiopia, even in its relatively modern and better-resourced capital city of Addis Ababa. Some recent progress in Ethiopia to scale up mental health services through the formal medical system by using task-shared care approach so less-specialized clinicians with proper training could care for the mentally ill (89), improving primary care capacity to care for people with mental illness (90), and working towards universal insurance coverage for mental illness (91), are showing some promise.

Nevertheless, recent studies in Ethiopia find the current formal system constitutes only a small part of overall system of care, and the available services are overwhelmed by demand and underwhelming in quality and sometimes under-attended (26, 82). An example of a solution to bridge these two separate modalities – between traditional healing and biomedical approach – is the Clinic where the study took place (48). The Clinic was freely accessible and provided much needed support to a very ill population, and in turn to their families. Overall, recent study and the current research show there is potential acceptance of biomedical treatment from patients and families, and even priests and attendants when the conditions of collaborations are well-thought out and respectfully implemented, focusing on mutual trust and acceptance (62, 92). Previous experience also show a cross-modality approach would work better if partners are not aiming to change each other’s professional practices, or displace any culturally ingrained, and practically beneficial approaches (48). Broadly speaking, there are many challenges in resource-limited settings to scale up mental health services. On the biomedical side, prioritizing mental health care, having sustained funding and resource allocation, and continued training are well reported challenges, among others (93–95). On collaborative approaches such as this current study setting, barriers often are related to the biomedical practitioners’ belief that the scientific model is superior, to the point of disregarding or devaluing traditional practices, and fearing that such collaboration would have the appearance of condoning or endorsing traditional practices (47). Issues of respect, acceptance, trust, discrimination are strong forces that contribute to a dearth of such collaborative work (44).

Lastly, the participants reported a strong sense of uncertainty, worry and hopelessness about future care of their ill family members. This is a culminating sentiment as they have already encountered great difficulty in accessing and affording care, and experienced present burden and diminishing support so far – to contemplate the future seemed unfathomable and dispiriting. These sentiments are notably similar to other research where formal services are wanting and reliance on family support is strong, compounded by low income, and poor quality of life, etc. (96–98). As many participants were parents, research also shows older caregivers experience more burden than younger ones, possibly related to their lengths of burden, state of health, and looming existential and financial uncertainties (99, 100). Through spirituality, having on-site help from attendants, king pride in their caring role, and creating mutual help communities, the participants showed general resilience amidst severe structural deficits, and did find some ways to cope with their hardship. Future service and intervention development and research could aim to improve these culturally meaningful areas to give support, maintain hope, empower the family caregivers, and aid in the recovery journey of the patients (3, 101).

## 5 Limitations

The current study focuses on understanding the family caregivers’ experiences and perspectives in their caring role. It is limited in data concerning the clinical functioning level or outcome of the patients they cared, and how that impacted on the families’ views. The sample size is relatively small and comes from one geographical location; therefore the representativeness and generalizability are limited. There may also be some self-selection bias as those who agreed to the study may over-represent those who were happy or not happy with their experiences. We also did not have enough sample size for finer understanding of how different relationships to patients may influence the caregiver experiences. The research topic may be a sensitive one for some, as not all may be open to talk about their sense of burden as it may be culturally expected to carry out their duty despite the hardship.

## 6 Conclusion

The current qualitative study of experiences and reflections of family caregivers for patients with severe mental illness in Holy Water treatment sites highlights that families in Ethiopia face many barriers to care, and endure hardships in familial and personal lives, with evident heavy caregiver burden. They also often suffer in isolation, with a severe lack of formal biological treatment and psychosocial support. Informal assistance and mutual support from fellow caregivers and Holy Water treatment community members form part of the culturally shaped support networks. Religious beliefs also help to give meaning and purpose in their hard work. Innovative programs with collaborative approach show some promise. Development of community mental health services and support for patients and family caregivers are urgently needed.

## Data Availability

The raw data supporting the conclusions of this article will be made available by the authors upon request, without undue reservation.
